# Effect of Korean nursing students’ experience of incivility in clinical settings on critical thinking

**DOI:** 10.1016/j.heliyon.2020.e04367

**Published:** 2020-07-11

**Authors:** Soon Ae Kim, Eunhee Hong, Gyun Young Kang, Cheryl Brandt, Younglee Kim

**Affiliations:** a Seoul Women's College of Nursing, 82 anhodae-Ro, Seodaemun- Gu, Seoul, South Korea; b Kyungdong University, Department of Nursing, 815 Gyeonhwon-ro, Munmak-eup, Wonju-si, Gangwon-do, South Korea; c California State University San Bernardino, Department of Nursing, 5500 University Parkway, San Bernardino, CA, 92407, USA

**Keywords:** Nursing, Clinical research, Health profession, Health promotion, Clinical practice, Critical thinking, Incivility, Nursing students

## Abstract

Clinical experience is essential to helping nursing students to achieve and practice professional knowledge and skills. Published reports indicate nursing students often experience incivility during clinical practice. The purpose of this study was to investigate nursing student incivility experience during their clinical rotations and the relationship of these experiences with their critical thinking dispositions. A cross-sectional descriptive correlational study design was utilized. Data were collected from junior (n = 195) and senior (n = 180) students in a Bachelor of Science in Nursing (BSN) program in Seoul, Korea between October 15, 2017 and November 20, 2017 using a self-administered survey. Three instruments were used in the survey: six sociodemographic questions, the 13-item Korean version of Uncivil Behavior in Clinical Nursing Education (K-UBCNE) and the 27-item Yoon Critical Thinking Disposition (YCTD) tool. Data analysis revealed junior students reported significantly more incivility from nurses than the senior students (p = .038) during clinical learning experiences. Among YCTD subscales, the Prudence (p = .007) and Self-Confidence critical thinking (p = .007) scores from the senior nursing students were significantly higher than the junior students’ scores. No significant relationship was found between experience of incivility and critical thinking disposition scores. Based on the study results, nursing educators, staff nurses, and administrators/managers in nursing should identify incivility toward nursing students during clinical practicums and especially toward junior nursing students. Additional investigation of the relationship between critical thinking and experiences of incivility is warranted, including longitudinal investigations and qualitative studies among junior nursing students to understand their personal experience of incivility in the clinical setting. Findings could inform the development of targeted programs to reduce clinical incivility.

## 1 Introduction

Clinical education is a necessary component of the nursing curriculum. Clinical practicums represent about 50% of a nursing curriculum; nursing students spend designated hours of clinical practice to complete the requirements of all nursing educational programs (Farzi et al., 2018). Through clinical education nursing students obtain opportunities to learn, practice, and improve professional skills (Brammer, 2006; Elcigil and Sari, 2007) and roles (Thomas et al., 2015), solve complex problems of health care, and apply knowledge and critical thinking skills learned from classes into real situations (Anthony et al., 2014; Orton, 2007). Therefore, clinical education is imperative to develop nursing practice knowledge (Thomas et al., 2015) as well as to maintain the quality and success of nursing programs.

One of the challenges students face in clinical education is incivility. According to the literature, incivility as a negative experience (Clark et al., 2010; Paul, 1990) refers to rude or destructive behavior that causes physical or psychological distress to others and creates threatening situations (Clark, 2009). Incivility may include verbal statements and/or any action or conduct that disrupts the work, social, personal, or educational environment (Sprunk et al., 2014). In academic environments incivility is a well-documented issue; victims of incivility become discouraged, with lower self-esteem and increased doubt about their abilities (Peters, 2015).

Documentation of clinical incivility is growing (Kolanko et al., 2006); it has become a big concern (Lewis et al., 2019; Meires, 2018; Etienne, 2014) as a global phenomenon in nursing practice and education (Birks et al., 2017). Nursing students practice in the same clinical settings as nurses but they are more vulnerable; nursing students have relatively less clinical experience than nurses and perform nursing care while adjusting to unfamiliar environments and people including patients, family members, and clinical site staff (Bowllan, 2015). Nursing students who experienced clinical incivility reported that they felt helpless (Anthony and Yastik, 2011), stressed (Anthony et al., 2014; Kim and Park, 2018), and less confident (Park and Kim, 2017). Bae and Im (2016) also found a negative relationship between incivility and students' learning process and outcomes, their ability to cope with situations, and the self-esteem they held regarding the nursing profession. In a study by Ziefle (2018), incivility toward students in the clinical setting had negative effects on their patients' nursing care practice. Incivility negatively affects students' goals of becoming professional nurses (Hyun et al., 2018). However, more studies of nursing students’ experience of clinical incivility are needed (Budden et al., 2017; Tecza et al., 2018).

Critical thinking is defined as the mental process of active and skillful perception, analysis, synthesis and evaluation of collected information through observation, experience and communication that leads to a decision for action (Papathanasiou et al., 2014). In the complex and constantly changing healthcare system, critical thinking including the ability to critique scientific evidence are regarded as vital requirements for education and professional practice (Kabeel and Eisa, 2016; Zuriguel Pérez et al., 2015). Critical thinking is required, along with creativity, to strengthen professional attitudes and behaviors and willingness of nurses to identify various potential problems in clinical situations (Chang et al., 2011; Papathanasiou et al., 2014). Thus, critical thinking is a key skill for nurses, enabling them to perform safe, effective, and skillful care based on scientific evidence (Paul, 1990; Sharifi et al., 2016).

In nursing education critical thinking has been accepted as a fundamental skill. The importance of critical thinking in nursing education and practice is globally recognized. Critical thinking can be developed and amplified by classroom exercises and clinical assignments (Papathanasiou et al., 2014). In other words, critical thinking may be developed through educational and practice activities such as decision making, problem solving, clinical reasoning and implementing the nursing process (Turner, 2005). During clinical practice, nursing students are required to explore and practice critical thinking (Maneval et al., 2011) while performing nursing actions to improve health outcomes such as satisfaction with care and patient safety (Naber et al., 2014). Clinical practicums provide nursing students with opportunities to think critically, apply information from theory classes, and reflect on their care experiences. As a result, students can develop critical thinking, reinforce self-knowledge, strengthen coping skills, and improve their clinical practice (Kennison, 2006). The Accreditation Commission for Education in Nursing (ACEN) in the United States (Accreditation Commission for Education in Nursing, 2019) and the Korea Accreditation Board of Nursing Education (KABNE) in South Korea (Korea Accreditation Board of Nursing Education, 2017) both include critical thinking as a necessary and crucial component for nursing education programs. However, there is limited research available to help understand the impact of the experience of incivility in clinical learning environments on nursing students’ critical thinking. Therefore, the objective of our study was to investigate relationships between nursing students' incivility experiences during clinical practicums and their critical thinking.

## 2 Methods

### 2.1 Study design and setting

Our study used a descriptive correlational design. We conducted a cross-sectional survey of nursing students enrolled in a Bachelor of Science in Nursing (BSN) program in a nursing college located in Seoul, the capital city of South Korea. The BSN program has a four-year program of study with approximately 850 students enrolled during the 2017–2018 academic year. About two hundred nursing students are admitted every year. In the first year of the BSN program, nursing students take liberal arts courses such as language or natural science classes and prerequisites such as anatomy or mathematics; they do not have clinical practicums either on campus or in hospitals. In the second year of the BSN program, nursing students complete 120 h of clinical practice in skills lab in the campus. During the third and fourth years nursing students take nursing core classes in medical-surgical nursing, maternal child and women's health, pediatric nursing, psychiatric/mental health nursing, and community health, attend campus lectures and engage in clinical practice at hospitals and in community settings. The students complete a total of 500 clinical hours during their third year and a total of 1000 h in hospitals and community healthcare settings during their fourth and final year.

### 2.2 Participants

The target population for our study was junior and senior nursing students. The junior students were in their third year of the BSN program in this nursing college and had already completed 450 clinical hours out of 500 h in hospitals, whereas the senior students were in the fourth year and had completed a total 750 clinical hours out of 1000 h. The study participants were all Koreans who spoke and wrote in the Korean language and were over 18 years old. They also had completed at least 150 clinical practicum hours in hospital settings. No participant self-reported having mental illness or taking any psychiatric medication or treatment.

### 2.3 Human subject approval

The South Korean University Institutional Review Board (IRB) approved the study proposal for use with human subjects (IRB No. 201708-HR-001-03). The study team met with the junior and senior nursing students who were prospective participants during a clinical practice session and described the research purpose, procedures, and participant expectations. Following the explanation, students completed the written study consent forms.

Completion of study surveys by consenting students took 20 min or less; to maintain anonymity, no personally identifying information was collected.

### 2.4 Data collection

Consenting junior and senior nursing students voluntarily completed the study survey between October 15, 2017 and November 20, 2017. A total of 425 nursing students began the survey but only 410 students completed it. The required sample size for our study was at least 153 participants based on a power analysis for an alpha of .05, power of .80 and a moderate effect size of .15 (G∗Power 3.1).

### 2.5 Instruments

The structured survey included six sociodemographic items (age, gender, year in the nursing program, marital status, employment, and religion), the 13-item Korean version of Uncivil Behavior in Clinical Nursing Education (K-UBCNE), and the 27-item Yoon Critical Thinking Disposition (YCTD) instrument.

#### 2.5.1 Incivility

Anthony and Yastik (2011) initially developed the UBCNE with 20 items, each with 5-point Likert response categories to measure nursing student experiences with incivility from nurses in the clinical learning environment. Later this instrument was modified and reduced to 12 items (Anthony et al., 2014). Jo and Oh (2016) reviewed the content validity, face validity, construct validity, and reliability of the original 20-item instrument by Anthony and Yastik (2011) and used their findings to develop the 13-item Korean version of the UBCNE (K-UBCNE) (Jo and Oh, 2016). The K-UBCNE includes three subscales: Hostile/Mean (H-M; 5 items), Exclusionary Behaviors (EXBEV; 5 items), and Dismissive (DIS; 3 items). Based on the five-point Likert style response categories (0 = never to 4 = very often) for each item, the 13-item total possible score is 0–52 with higher scores representing more experiences with incivility. Cronbach's alpha estimates of internal consistency reliability of the full 13-item K-UBCNE (α = .84) and the three subscales of H-M (α = .79), EXBEV (α = .77), and DIS (α = .68), were calculated using data from the study by Jo and Oh (2016). In our study the Cronbach's alpha for the K-UBNE total test was .91 and from .78 to .88 for the three subscales, H-M, EXBEV, and DIS.

#### 2.5.2 Critical thinking (CT)

To measure critical thinking, the participants completed the YCTD developed by Yoon (2004), based on the California Critical Thinking Disposition Inventory (CCTDI), for use with Korean nursing students. The YCTD has 27 items and uses 5-point Likert type response categories (1 = strong disagreement to 5 = strong disagreement). The instrument has the following seven subscales: Healthy Skepticism (4 items), Objectivity (3 items), Systematicity (3 items), Prudence (4 items), Intellectual Eagerness/Curiosity (5 items), Intellectual Fairness (4 items), and Self-Confidence (4 items). Thus total possible scores ranged from 27 to 135; the higher the score, the stronger the disposition towards critical thinking. Reliability and validity were verified by Yoon (2004) in the process of developing the instrument. The validity of the YCTD was reaffirmed by using cross-sectional and longitudinal surveys of Korean nursing students and employing multigroup confirmatory factor analysis (Shin et al., 2015). Cronbach's alpha estimates of internal consistency reliability were .84 for the YCTD total; Cronbach's alpha for the seven subscales ranged from .80 to .90 (Yoon, 2004). In our study the Cronbach's alphas were .87 for the YCTD total and ranged from .78 to .88 for the three subscales.

### 2.6 Statistical analysis

The research team used the IBM Statistical Package for Social Sciences (SPSS) version 24 for data analysis. Descriptive statistical tests included frequency counts, percentages, and mean scores (M) with standard deviations (SD) for the general characteristics of the sample. The sample was divided into two groups by junior and senior year in the BSN program. Then chi square analysis was performed to investigate sociodemographic characteristic differences between them.

Using independent t-test and Analysis of Variance (ANOVA), the differences in Incivility and CT scores were calculated by sociodemographic variables (age, year of nursing program, marital status, employment, and religion). Pearson correlation coefficients were calculated to examine the correlations among the Incivility total and subscale scores (H-M, EXBEV, and DIS), and CT total and subscale scores (Healthy Skepticism, Objectivity, Systematicity, Prudence, Intellectual Eagerness/Curiosity, Intellectual Fairness, and Self-Confidence).

## 3 Results

### 3.1 Sample characteristics

A total of 410 nursing students in South Korea completed the survey. They were all female. Among the 410 participants, over 90% (n = 375) reported that they had experienced incivility during their clinical practice. The data analysis for our study was conducted using the survey responses from the 375 nursing students who reported having experienced clinical incivility. Table 1 describes the sample characteristics (n = 375) in detail. The mean age of the junior group (n = 195) was 26.24 years (SD = 10.28) whereas the senior group (n = 180) was 24.40 years (SD = 9.12). Chi-square analysis determined no significant difference in the demographic characteristics (age category, marital status, employment, & religion) between the junior and senior students.

**Table 1:** Participants Demographics (N = 375).

Variable	Junior student *n* (%)	Senior student *n* (%)	χ^2^
Total sample	195 (52.0)	180 (48.0)	
Age			
Mean age ±SD	26.24 ± 10.28	24.40 ± 9.12	
20-30	186 (95.38)	176 (97.78)	.206
Over 30	9 (4.62)	4 (2.22)	
Marital status			
Married	182 (93.33)	172 (95.56)	.350
Single	13 (6.67)	8 (4.44)	
Employment			
Unemployed	149 (76.41)	128 (71.11)	.243
Employed	46 (23.59)	52 (28.89)	
Religion			
No religion	125 (64.11)	113 (62.78)	.769
Protestant	39 (20.00)	40 (22.22)	
Catholic	26 (13.33)	20 (11.11)	
Buddhism	5 (2.56)	7 (3.89)	

### 3.2 Associations between incivility and critical thinking dispositions

In this study, for the entire sample (n = 375) the mean scores of Total Incivility measured by the K-UBCNE and total CT measured by the YCTD were 25.35 (SD = 9.79) and 98.55 (S = 10.28). No significant association was found between the Total Incivility score and the Total CT score. However, the junior students' Incivility mean score (M = 26.24, SD = 10.30) was significantly higher than the senior students’ mean score (M = 24.40, SD = 9.17; t = 1.828, p = .038). Among three subscales of the K-UBCNE for incivility, the H-M (t = 3.233, p = .001) and DIS (t = 4.425, p = .001) mean scores of the junior nursing students were significantly higher than those of the senior students.

Regarding the Total CT score as measured by the YCTD, compared to the seniors' mean score (M = 100.20, SD = 10.78) the juniors' mean score was lower (M = 98.11, SD = 9.96), though the difference was not significantly different (t = -1.53, p = .126). Among the seven subscales of the CT, both junior students (M = 17.71, SD = 2.82) and senior students (M = 17.66, SD = 3.13) scored highest on Intellectual eagerness/Curiosity. Systematicity was the lowest score for both juniors (M = 10.19, SD = 1.66) and seniors (M = 10.33, SD = 1.91). The juniors had higher mean subscale scores in two of the seven categories, Healthy Skepticism and Intellectual Eagerness/Curiosity; however, the differences between the two groups were not significant. By contrast, the junior and senior students' mean scores on two of the seven CT subscale scores were significantly different: Prudence (t = - 2.744, p = .007) and Self-Confidence (t = - 2.795, p = .006). In both instances, the juniors' mean scores was approximately one point lower than the seniors’ (see Table 2 for the details).

**Table 2:** South Korean junior and senior nursing student clinical incivility and critical thinking scores (N = 375).

Instruments a	Juniors	Seniors	*t*	*p*^b^	95% CI	
	M (SD)	M (SD)			Lower	Upper
Total Incivility (K-UBCNE)	26.24 (10.30)	24.40 (9.17)	1.828∗	.038	-.14	3.80
Hostile/Mean	12.23 (4.55)	10.07 (4.49)	3.233∗∗	.001	-.12	1.54
Exclusionary Behaviors	11.05 (4.28)	10.34 (3.88)	.815	.416	-.77	1.07
Dismissive	5.10 (2.72)	3.98 (2.48)	4.425∗∗	.001	.45	1.51
Total Critical Thinking (YCTD)	98.11 (9.96)	100.20 (10.78)	-1.534	.126	-5.30	-1.26
Healthy skepticism	14.62 (2.21)	14.39 (2.36)	.761	.447	-.59	.31
Objective	12.11 (1.33)	12.42 (1.48)	-1.092	.279	-.56	-.02
Systematicity	10.19 (1.66)	10.33 (1.91)	.083	.540	-.61	.10
Prudence	13.73 (2.39)	14.58 (2.31)	-2.744∗∗	.007	-1.46	-.51
Intellectual eagerness/curiosity	17.71 (2.82)	17.66 (3.13)	.120	.905	-.71	.67
Intellectual fairness	15.44 (2.02)	15.81 (2.09)	-1.373	.171	-.95	-.17
Self-confidence	14.19 (2.34)	15.00 (2.03)	-2.795∗∗	.006	-1.29	-.40

a K-UBCNE: Korean version-uncivil behavior in clinical nursing education, YCTD: Yoon's critical thinking disposition.

b ∗p < .05 (2-tailed), ∗∗p < .01 (2-tailed).

### 3.3 Correlations among all variables

Pearson correlation coefficients were computed between the total incivility and total critical thinking scores, the total critical thinking score and the three incivility subscale scores (H-M, EXBEV, and DIS), and between the total incivility score and the seven critical thinking subscale scores (Healthy Skepticism, Objective, Systematicity, Prudence, Intellectual Eagerness/curiosity, Intellectual Fairness, and Self-Confidence). Additionally, Pearson correlation coefficients were computed among the three incivility subscale scores and the seven critical thinking subscale scores. No statistically significant relationships were found.

## 4 Discussion

Among this sample of 410 South Korean nursing students, a total of 375 students (91.46%) had experienced incivility during their clinical learning experiences. This finding is similar to that of Hong et al. (2016) with another Korean sample of nursing students; in their study approximately 97 % (n = 117 of 120) reported experiencing incivility in clinical settings. In a sample of nursing students in Australia, 50% (n = 446 of 888) reported bullying or harassment during their clinical practice (Budden et al., 2017). From a study of 126 baccalaureate nursing students in Canada, 59 % reported experiencing incivility in clinical practice settings (Babenko-Mould and Laschinger, 2014). Authors of another study in Australia also reported that more than 50 % of nursing students (n = 76 of 152) experienced incivility during their clinical rotation. In a United Kingdom study, about 53% (n = 165 of 313) reported experiencing incivility during clinical rotations (Stevenson et al., 2006).

In a study of 833 Australian sample and 561 UK sample, Birks et al. (2017) found that 417 Australian nursing students (50.1%) and 199 UK nursing students (35.5%) experienced incivility in clinical placements. Babenko-Mould and Laschinger (2014) also reported that 59 % (n = 74 of 126) experienced clinical incivility from nurses. Nurses were the source and main perpetrators of incivility to nursing students in clinical areas. This body of evidence highlights the prevalence of nursing students’ experience with clinical incivility during their education experience.

Nurses practicing at clinical sites at which nursing students are placed are very important and influential as key educators and supporters of students' clinical learning and practice (Luparell, 2011). Thus nurses should recognize the impact of their incivility behaviors; these behaviors can significantly influence nursing students’ burnout (Laschinger et al., 2009) as well as result in loss of motivation for learning, low self-esteem, and feelings of incompetence (Hakojärvi et al., 2014).

Clinical instructors are also important for nursing students' education. Clinical instructors often conduct periodic assessments to identify incivility toward nursing students; results should be reported to administrators of both the school and the clinical area. It is the responsibility of both clinical instructors and practicing nurses to be aware of the risk for incivility toward nursing students (Altmiller, 2012; Del Prato, 2013) and to take action to create and maintain positive learning environments in clinical education settings (Clark, 2009; Clark et al., 2015). Furthermore, clinical nurse administrators should reinforce the responsibility of nurses as well as other healthcare staff to contribute to nursing students’ educational achievements through respectful behaviors in clinical sites (Anthony and Yastik, 2011). Academic institutions should assist nursing students to accurately address their incivility experiences and educate academic staff and faculty to appropriately respond incivility in clinical learning environments (Minton and Birks, 2019). Nursing students also need to be prepared for the possibility of encountering incivility in clinical sites. Faculty and clinical instructors should introduce nursing students to the literature and provide information about appropriate responses to experiences of incivility (Thomas, 2015). Students should be encouraged to report incivility to clinical instructors as well as meet with counselors to address their negative feelings. Follow-up care can be offered based on student need.

In our study the junior nursing students reported significantly more incivility experiences than the senior nursing students. In particular, on three Incivility subscales the juniors reported experiencing more incidents of Hostile/Mean and Dismissive incivility than did the seniors. This finding is similar to the finding by others of a significant inverse association between length of clinical experience and nursing students’ perception of incivility in clinical settings (Mott, 2014; Rawlins, 2017). These findings strengthen the need for clinical instructors to discuss with students before their first clinical rotation the possibility of encountering incivility as well as how they might respond and how to report it (Thomas, 2015).

Helpful information about educational interventions to help students learn about and manage incivility has been described in a paper by Rutherford et al. (2019). Rutherford et al. (2019) conducted an integrative review of qualitative, quantitative, mixed-methods, and quality improvement studies of bullying of prelicensure nursing students. Five educational interventions for incivility were identified in the course of the review; they were a) curriculum integration, b) problem-based learning (PBL), c) journaling, d) cognitive rehearsal, and e) educational courses within the health care settings. Curriculum integration was carried out to provide nursing students with information and knowledge about incivility and that it represents unprofessional behavior (Rutherford et al., 2019). The PBL intervention was implemented to moderate the effects of incivility or bullying behaviors. Nursing students were provided with a clinical scenario of incivility and through problem-based learning strategies including self-directed learning as well as group discussions were able to develop positive strategies to effectively prevent and manage their incivility experience (Clark et al., 2014). Journaling is to record personal experience and perception of incivility (Jenkins et al., 2013). Journaling can provide an opportunity to review and understand situations and problems related to incivility and to change personal perception of incivility (Jenkins et al., 2013). Lastly, cognitive rehearsal has been employed as a mental recognition strategy to positively rethink of negative incivility experience (Razzi and Bianchi, 2019). These interventions should be considered for use with nursing students, in particular with junior students, to prevent and manage clinical incivility in clinical sites.

Interestingly, according to our study results there was no significant difference in mean total scores on the YCTD between the junior group and senior group. By contrast, Jung (2011) found that senior students' total Critical Thinking mean score on the YCTD was significantly higher than that of the junior group (F = 2.700, p = .047) in a comparative study among Korean nursing students. In considering possible explanations for the difference in our findings from those of Jung (2011), it is noted that our study sample included only female junior and senior nursing students, while Jung's study also included both male and female freshman and sophomore nursing students. Additionally, Jung's sample size (n = 228) was smaller than our sample (n = 375); the larger sample may have helped reduce potential bias.

Jung (2011) also found that among all seven subscales in the YCTD, Intellectual eagerness/Curiosity was the highest score in the junior (17.61 ± 2.95) and senior (17.63 ± 2.67) students. Only on the Intellectual Fairness subscale (F = 3.062, p = .029) did Jung (2011) find a significant subscale score difference between the juniors and seniors. In our study the seniors’ Prudence and Self-confidence mean scores were significantly higher than those of the junior students. The items in these subscales asked the students to evaluate whether they could carefully respond to and confidently handle complicated problems during their clinical practice. Similar to our study findings, other researchers also indicated that less clinical experience was related to lower self-confidence (Makarem et al., 2019; Manomenidis et al., 2017) which can make a negative effect on safety and quality of patient care in hospitals (Pfaff et al., 2014). Therefore, education to prevent incivility for nursing students should include exercises to improve their prudence and self-confidence for successful clinical education achievement.

In our study sample, mean scores on Incivility as measured by the K-UBCNE and Critical Thinking as measured by the YCTD were not significantly related. A search of international studies published from 2008 through 2018 using similar measures yielded 12 studies of Korean nursing students. Hong et al. (2016), Jeon and Oh (2017), and Kim et al. (2017) found significant positive correlations between incivility toward students and their feelings of burnout in clinical practice. In a sample of 210 nursing students, Jeon and Oh (2017) also found that incivility experience scores were negatively correlated with self-efficacy during their clinical practice. Kim et al. (2017) also reported significant positive correlations between the experience of incivility and coping among 160 nursing students in clinical settings.

Likewise, two studies reported significant positive relationships of critical thinking, measured with the YCTD, with problem solving ability and self-directed learning in 167 Korean nursing students (Choi and Park, 2014). Kim et al. (2014) found increases in YCTD critical thinking scores following a scenario-based learning program for nursing students. However, none of these studies investigated the relationship of incivility and critical thinking among nursing students during clinical practice.

Ultimately, our study found no statistically significant relationships between the total incivility and critical thinking scale scores, the total critical thinking and the three incivility subscale scores, (H-M, EXBEV, and DIS), the total incivility and the seven critical thinking subscale scores (Healthy Skepticism, Objective, Systematicity, Prudence, Intellectual Eagerness/curiosity, Intellectual Fairness, and Self-Confidence), nor among the incivility and critical thinking subscale scores. Our findings do not point to the existence of a relationship between critical thinking and clinical incivility experiences in nursing students. We were unable to find other studies investigating this relationship with which to compare our findings. Because the relationship between these two constructs has not been well-studied, additional investigation is warranted. Our study was cross-sectional; longitudinal investigation of the impact of incivility experiences on critical thinking over time would be of interest. Our study only investigated the relationship between the experience of incivility and critical thinking in nursing students enrolled in a single nursing college. The findings should not be generalized to other nursing students from various schools and locations. Future research about nursing student encounters of incivility during their clinical experiences should include assessment of other factors such as learning satisfaction, self-efficacy, or communication skills. As noted above, a longitudinal investigation of the impact of incivility experiences on critical thinking would be informative. Qualitative methods could be used to gather in-depth information from individual students who have experienced incivility and the influence these experiences have had on the development of their critical thinking. Interviews would also identify experiences of incivility experienced with other healthcare providers/staff or patients’ family members.

## 5 Conclusions

Incivility has a negative effect on clinical education. Our study findings are consistent with reports in the literature about the prevalence of incivility experienced by nursing students during their clinical at the hands of nurses. This study contributes to the knowledge of the impact of incivility experienced during the formative nursing student years. Educators and nurses should recognize the seriousness and potential negative impacts of incivility in clinical settings and find effective methods to assist nursing students. Further in-depth research on incivility directed against nursing students in clinical practicums and on nurses’ awareness of incivility should be conducted. Based on these findings, incivility prevention measures can be developed to include educational exercises for students and professional development programs for the nurses who interact with them.

## Declarations

### Author contribution statement

Soon Ae Kim, Eunhee Hong: Conceived and designed the experiments; Performed the experiments; Analyzed and interpreted the data; Contributed reagents, materials, analysis tools or data; Wrote the paper.

Gyun Young Kang, Younglee Kim: Conceived and designed the experiments; Contributed reagents, materials, analysis tools or data; Wrote the paper.

Cheryl Brandt: Wrote the paper.

### Funding statement

This study did not receive any specific grant from funding agencies in the public, commercial, or not-for-profit sectors.

### Competing interest statement

The authors declare no conflict of interest.

### Additional information

No additional information is available for this paper.
